# Value of automatic patient motion detection and correction in myocardial perfusion imaging using a CZT-based SPECT camera

**DOI:** 10.1007/s12350-016-0571-7

**Published:** 2016-07-12

**Authors:** Joris D. van Dijk, Jorn A. van Dalen, Mohamed Mouden, Jan Paul Ottervanger, Siert Knollema, Cornelis H. Slump, Pieter L. Jager

**Affiliations:** 1 Department of Nuclear Medicine, Isala Hospital, PO Box 10400, 8000 Zwolle, GK The Netherlands; 2 Department of Medical Physics, Isala Hospital, Zwolle, The Netherlands; 3 Department of Cardiology, Isala Hospital, Zwolle, The Netherlands; 4 MIRA: Institute for Biomedical Technology and Technical Medicine, University of Twente, Enschede, The Netherlands

**Keywords:** Myocardial perfusion imaging (MPI), cadmium-zinc-telluride (CZT), patient motion, respiratory motion, motion correction

## Abstract

**Background:**

Correction of motion has become feasible on cadmium-zinc-telluride (CZT)-based SPECT cameras during myocardial perfusion imaging (MPI). Our aim was to quantify the motion and to determine the value of automatic correction using commercially available software.

**Methods and Results:**

We retrospectively included 83 consecutive patients who underwent stress-rest MPI CZT-SPECT and invasive fractional flow reserve (FFR) measurement. Eight-minute stress acquisitions were reformatted into 1.0- and 20-second bins to detect respiratory motion (RM) and patient motion (PM), respectively. RM and PM were quantified and scans were automatically corrected. Total perfusion deficit (TPD) and SPECT interpretation—normal, equivocal, or abnormal—were compared between the noncorrected and corrected scans. Scans with a changed SPECT interpretation were compared with FFR, the reference standard. Average RM was 2.5 ± 0.4 mm and maximal PM was 4.5 ± 1.3 mm. RM correction influenced the diagnostic outcomes in two patients based on TPD changes ≥7% and in nine patients based on changed visual interpretation. In only four of these patients, the changed SPECT interpretation corresponded with FFR measurements. Correction for PM did not influence the diagnostic outcomes.

**Conclusion:**

Respiratory motion and patient motion were small. Motion correction did not appear to improve the diagnostic outcome and, hence, the added value seems limited in MPI using CZT-based SPECT cameras.

**Electronic supplementary material:**

The online version of this article (doi:10.1007/s12350-016-0571-7) contains supplementary material, which is available to authorized users.

## Introduction

Myocardial perfusion imaging (MPI) is known as one of the best validated noninvasive methods to test for ischemia,^1^ although artefacts negatively influence the clinical accuracy. Introduction of patient-specific activities and CT-based attenuation correction have limited this influence.^2^–^4^ However, artefacts still occur and may mainly be the result of motion.^5^,^6^ Artefacts resulting from patient movement, respiration, and myocardial contraction are difficult to distinguish from real perfusion defects and can lead to false positive studies.^6^–^10^

Several types of motion tracking and techniques to correct for overall patient motion (PM) on conventional SPECT cameras have been introduced and validated.^9^,^11^,^12^ However, these techniques cannot be applied in SPECT cameras equipped with stationary cadmium zinc telluride (CZT) detectors with pinhole collimators. Two recent studies showed that motion detection and correction seems feasible on a CZT-based SPECT camera.^8^,^13^ However, they did not compare their motion correction to a reference standard. It is therefore unknown whether the corrections improved the diagnostic outcomes.

A commercially available automatic motion detection and correction software has become available which can detect and correct for both respiratory motion (RM) and PM in all directions using a CZT-based SPECT camera. This program (MCD for Alcyone, GE Healthcare) has—to the best of our knowledge—not been described or validated in clinical practice before. Hence, the aim of our study was to quantify the motion and to determine the value of automatic correction using commercially available software.

## Methods

### Study Population

We retrospectively included 83 consecutive patients in this study who underwent clinically indicated elective FFR measurement of intermediate anatomical coronary lesions demonstrated by recent invasive angiography between January 2011 and July 2014.^14^ One day after the FFR measurement, patients underwent CZT-SPECT/CT 1-day stress-rest MPI (Discovery NM/CT 570c, GE Healthcare). All patients provided written informed consent for the use of their data for research purposes. Inclusion criteria were referral for elective FFR measurement of target lesions with a reduction in diameter of 40% to 80% as determined during previous coronary angiography, conform a previous study using the same population.^14^ Patients with concomitant severe coronary stenosis (>80%), serial coronary stenosis, and prior myocardial infarction in the territory of the target lesion or for whom list mode data were missing were excluded.

### CZT-SPECT Data Acquisition

Patients were instructed not to use any nicotine or caffeine containing beverages for 24 hours and to discontinue persantin for 48 hours prior to scanning. Pharmacological stress was induced by intravenous adenosine (140 μg·kg^−1^·minute^−1^ for 6 minutes) or dobutamine (starting from 10 μg·kg^−1^·minute^−1^, increased along three minute intervals to a maximum of 50 μg·kg^−1^·minute^−1^ until 85% of the predicted maximum heart rate was reached). Only pharmacologic stress was used due to logistic reasons, in particular the high patient throughput in our center.^15^ At peak stress, patients were injected intravenously with 370 MBq (10 mCi) Tc-99m tetrofosmin (500 MBq (13.5 mCi) for patients with a body weight of more than 100 kg). Rest imaging was performed on the same day using 740 MBq (20mCi) Tc-99m tetrofosmin.

Patients were scanned in supine position, with arms placed above their head using a fixed scan time of 8 minutes for the stress acquisition. The patient’s chest was positioned close to the SPECT detectors, with the heart in the center of the field of view, assisted by using real-time persistence imaging. Both stress and rest acquisitions were performed 45-60 minutes post injection using a 20% symmetrical energy window centered at 140 keV. Data were acquired in list mode. Attenuation correction was not used in this study to prevent reproducibility errors.^16^ A full description of the CZT detector system used in this study is described in several studies.^15^,^17^–^19^ In short, the scans were acquired using 19 stationary pinhole detectors, each containing 32 × 32 pixelated (2.46 × 2.46 mm) CZT elements, all focused on the myocardium.

### Motion Detection and Correction

All emission data were reformatted into 1.0- and 20-second time bins for RM and PM detection and correction, respectively. Next, a volume of interest was drawn manually around the myocardium to exclude extra cardiac activity. Motion was tracked by commercially available software (MDC for Alcyone, Xeleris 3.1, GE Healthcare). In short, the algorithm determines the center of mass in the detected counts for five pinhole projections in the user-defined volume of interest. Next, five virtual lines originating from these center of mass’ are drawn through these pinholes, and the point (*x*,*y*,*z*) with the smallest distance from these lines is calculated. This process is repeated for each time bin, and afterwards all points are compared to identify motion. The magnitude of RM was only assessed in the *z*-direction, caudal-cranial, as this is the main contributor to respiratory motion.^8^ PM was assessed in all three directions: lateral motion, ventral-dorsal motion, and cranial-caudal motion. Overall PM was estimated by calculating the square root of the summed squared motions in all three directions for each time bin. All motions were automatically corrected using the same software by generating a system matrix that incorporated the identified motion which was then used to reconstruct the images from the original projections.

### CZT-SPECT Reconstruction

The noncorrected and motion-corrected images were reconstructed by applying an iterative dedicated reconstruction algorithm with maximum-likelihood expectation maximization (Myovation, Xeleris 3.1, GE Healthcare). The use of dedicated software (Make SA and Fil3DBatch, Xeleris 3.1, GE healthcare) allowed to reconstruct both the noncorrected and motion-corrected images using the exact same alignment and masking, excluding possible reproducibility errors.^16^

Each image was automatically normalized to the maximum peak activity and the 17-segmental uptake values were presented as the percentage of the maximum myocardial regional uptake. Total perfusion deficit (TPD) was automatically calculated for all scans (Quantitative Perfusion SPECT (QPS) 2009, Sedar Senai). TPD is defined as the percentage of segments below the predefined uniform average deviation threshold, as explained in detail by Berman et al.^20^ Scans were displayed in the traditional short, vertical long, and horizontal long axes and reviewed using a color scale.

### FFR Measurements

FFR measurements were derived in the same way as previously described.^14^ In short, we introduced a pressure monitoring wire (PressureWire®; RADI Medical Systems) into the coronary artery and positioned the pressure wire distal to the stenosis. Adenosine (140 μg·kg^−1^·minute^−1^) was infused continuously to obtain a maximal coronary blood flow. The FFR was calculated by dividing the mean distal intracoronary pressure by the mean arterial pressure proximal of the possible stenosis. FFR ratios <0.80 were considered positive for ischemia and FFR ratios ≥0.80 were considered negative for ischemia.^21^

### Added Value of Motion Correction

The mean and maximum RM and PM were derived from all time bins. The mean RM and PM across all patients during the scan were assessed to determine a possible increase in motion with longer scan times. Next, the noncorrected scans were compared with the RM-corrected scans and also with the PM-corrected scans to determine the possible change in diagnostic outcome.

In the qualitative evaluation, two experienced readers interpreted in consensus whether there was a change in diagnostic outcome (categorized as normal, equivocal, or abnormal) between the noncorrected and motion-corrected scans. Readers were aware which series were noncorrected or motion-corrected but they had no knowledge of patients’ history or other clinical findings. In the quantitative evaluation, the differences in TPD and segmental uptake values were determined between the noncorrected and corrected scans. A difference in TPD of ≥7% was considered to result in a change in diagnostic outcome, as previously described by Berman et al and Iskandrian et al.20,^22^ A difference of ≥5% in at least one segment was also considered to affect the diagnostic outcomes as it is associated with mild to severe ischemia.^23^,^24^

Next, we compared the conclusion of the scans in which the diagnostic outcome was changed after motion correction with the FFR measurements to determine whether motion correction resulted in a better correspondence with FFR. Concordance with SPECT interpretation was determined on a per-vessel basis by comparing the changed perfusion in the area supplied by the vessel with the FFR measurement performed in that vessel.

### Statistics

Patient-specific parameters and characteristics were determined as mean ± SD using Stata (StataSE, version 12.0). The correlation between the amount of movement and scan time was assessed using the Pearson correlation coefficient. Correlations between the detected motion and change in visual SPECT interpretation and number of segments differing ≥5% were tested using the Spearman rank correlation coefficient. The correlation between the amount of motion and change in TPD was assessed using the Pearson correlation coefficient. The level of statistical significance was set to .05 for all statistical analyses.

## Results

The baseline characteristics of all included patients are summarized in Table 1.

**Table 1:** Baseline characteristics and scan outcomes of all patients who underwent clinically indicated MPI SPECT

Characteristic	(n = 83)
Age (years)	66.6 ± 10.5
Male gender (%)	41.0
Body weight (kg)	85.3 ± 14.5
Height (cm)	174 ± 8.7
BMI (kg·m^−2^)	28.2 ± 4.4
Current smoking (%)	18.1
Hypertension (%)	65.1
Diabetes (%)	21.7
Dyslipidemia (%)	63.9
Family history (%)	59.0
Normal MPI scan (%)	71.2
Ischemic defect on MPI (%)	20.5
Nonreversible defect on MPI (%)	19.3
Summed stress score	4.1 ± 8.1
Total perfusion deficit (%)	4.3 ± 7.9

Data are presented as mean ± SD or percentages

### Motion Detection

RM and PM were observed in all 83 patients. The mean RM, in cranial-caudal direction only, was 2.5 ± 0.4 mm, as shown in Table 2. The maximum PM across all patients in all three directions were 2.4 ± 0.8 mm, 2.8 ± 0.9, and 3.4 ± 1.5 mm in the lateral, ventral-dorsal, and cranial-caudal direction, respectively.

**Table 2:** Mean and maximal respiratory motion and patient motion in MPI CZT-SPECT for all included patients (n = 83)

Characteristic	Mean motion	Maximum motion
Absolute respiratory motion		
Cranial-caudal (mm)	2.5 ± 0.4 (1.7–3.7)	10 ± 2.0 (6.0–15.3)
Absolute patient movement		
Lateral (mm)	0.9 ± 0.2 (0.3–1.5)	2.4 ± 0.8 (0.9–5.4)
Ventral-dorsal (mm)	1.0 ± 0.2 (0.0–1.8)	2.8 ± 0.9 (0.0–5.7)
Cranial-caudal (mm)	1.2 ± 0.5 (0.5–2.5)	3.4 ± 1.5 (1.1–8.9)
Overall (mm)	2.1 ± 0.4 (1.2–3.2)	4.5 ± 1.3 (2.6–9.3)

Data are presented as mean ± SD and the ranges are shown between parentheses

**Figure 1:**
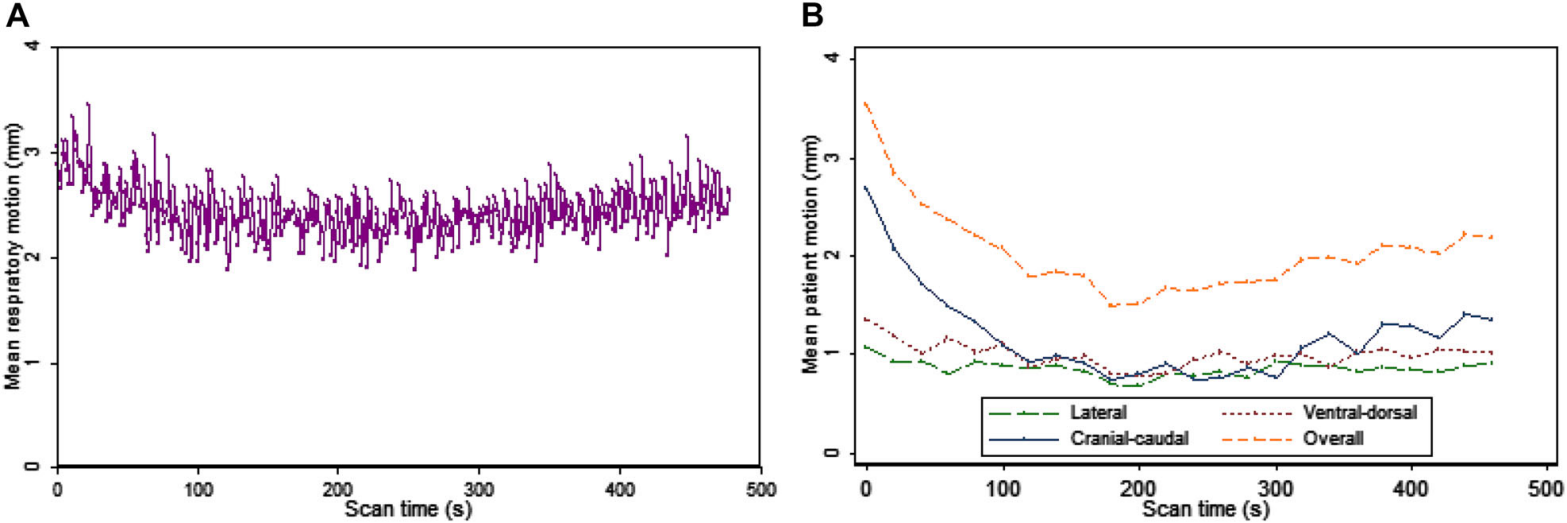
The mean (**A**) respiratory motion in the cranial-caudal direction and (**B**) patient motion in all three directions and the overall patient motion as function of scan time. The respiratory motion decreased significantly during the 8-minute acquisition (*P* = .01), whereas this was nearly significant for the overall patient motion (*P* = .06)

The mean RM across all patients decreased significantly during the scan (*P* = .01). Especially in the first 2 minutes the RM decreased and seemed to stabilize or slightly increase again after 4 minutes, as shown in Figure 1. A similar trend was observed for the mean PM, although this was not significant (*P* = .06).

### Diagnostic Value of Respiratory Motion Correction

**Figure 2:**
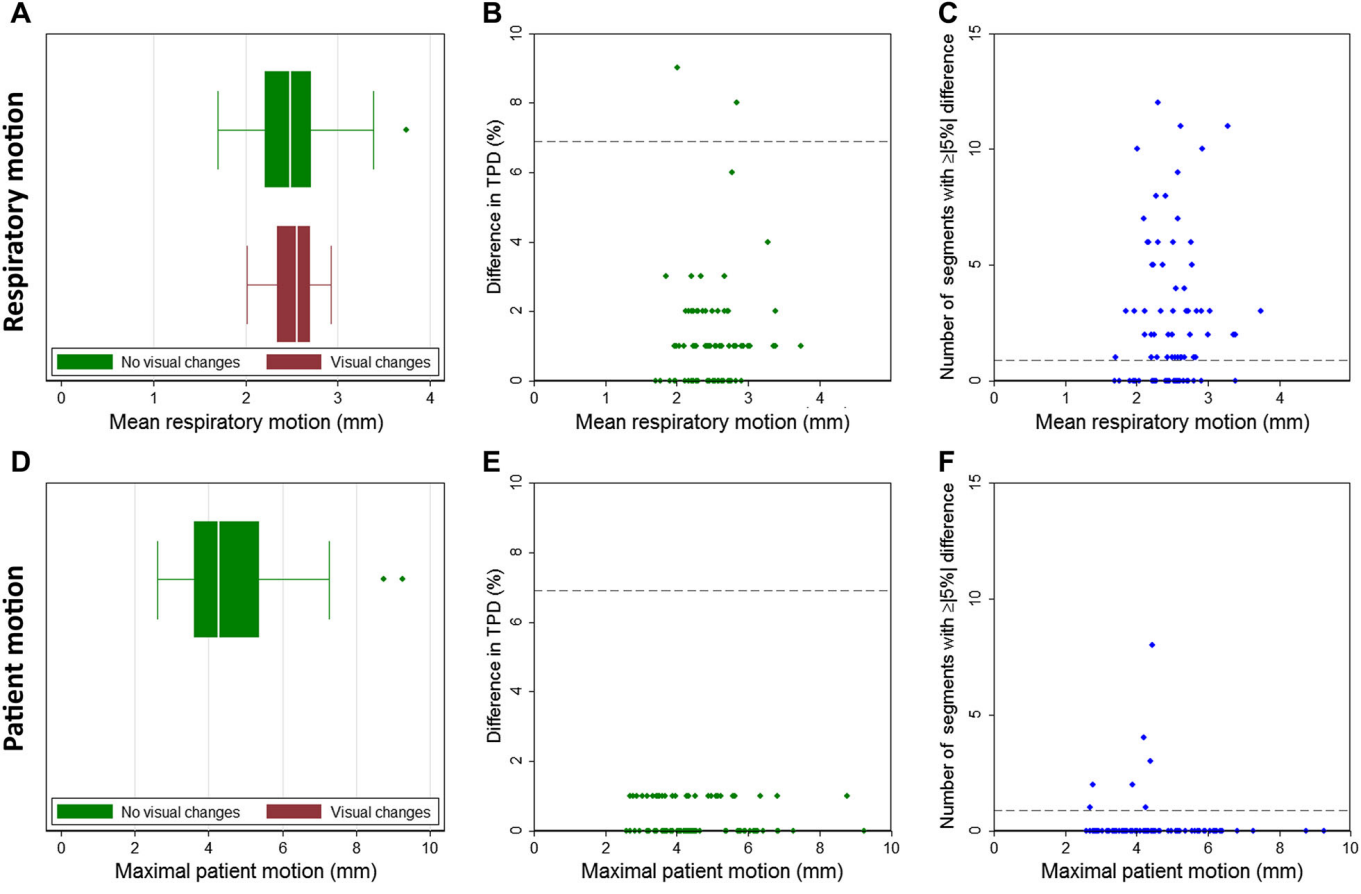
Relation between both respiratory (*top row*) and patient motion (*bottom row*) and the differences between the noncorrected and motion-corrected scans in (**A**, **D**) the visual SPECT interpretation, (**B**, **E**) the total perfusion deficit, and (**C**, **F**) the number of changed segments ≥5%. Neither patient motion nor respiratory motion correlated with the differences in one of the three endpoints (*P* > .26). Note that no changes in SPECT interpretation were found after correction for patient motion (**D**). The *dashed lines* represent the thresholds for which the diagnostic outcome was considered to be influenced

The visual SPECT interpretation remained unchanged after RM correction in 74 (89%) patients but changed in nine patients (11%) after applying RM correction, as shown in Figure 2A. These changes were in correspondence with FFR in only four of these nine patients: the SPECT interpretation changed from normal to equivocal in six patients (7%) corresponding with FFR in two. So, in these two patients, the SPECT result was closer to the reference standard and the motion correction could therefore be considered a slight improvement. However, in four out of these six patients, the change could be considered a deterioration. The SPECT interpretation changed from abnormal to equivocal in two of the nine patients. In these two patients, a positive FFR was measured in one (0.65)—considered a deterioration—and a negative FFR was measured in the other patient (0.85), considered an improvement. In the remaining patients, the SPECT interpretation changed from equivocal to normal, which was an improvement as it corresponded with the negative FFR measurement (0.88). In summary, motion correction produced a total of four improvements and five deteriorations in the overall diagnostic outcome of the SPECT study based on visual interpretation.

**Figure 3:**
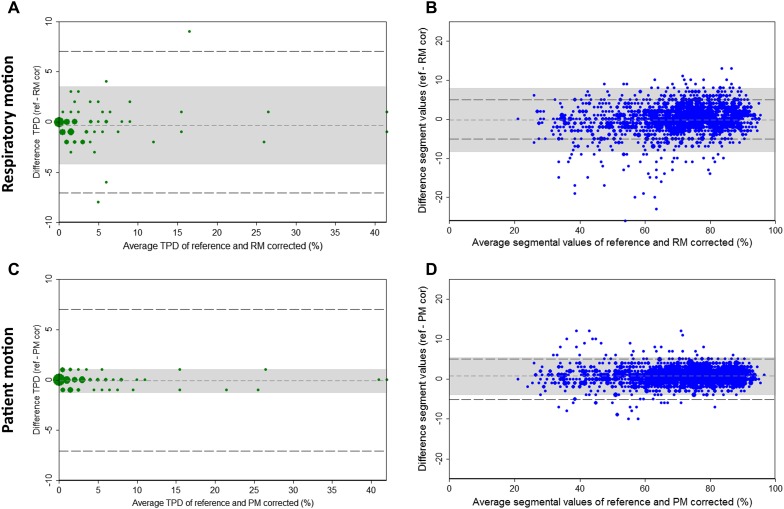
Bland Altman plots of the noncorrected and motion-corrected images for (**A**, **B**) the respiratory motion (RM) and (**C**, **D**) the patient motion (PM). The *left images* (**A**, **C**) show the differences in total perfusion deficit (TPD) and the *right images* (**B**, **D**) show the differences in segmental uptake values of all 17 segments of all patients. The *shaded area*s represent the 95% confidence interval limits and the *long dashed lines* represent the thresholds for which the diagnostic outcome was considered to be influenced

By analyzing the impact of RM correction using TPD as a semiquantitative parameter, we found a ≥7% change in TPD after RM correction in only two patients (3% of the whole group), as shown in Figure 3A. In one patient, the TPD increased from 1% to 9% which was considered an improvement as the FFR was positive (0.77). In the other patient, the TPD decreased from 21% to 12% which was considered a deterioration as the FFR was positive (0.65). So, RM correction resulted in one improvement and one deterioration in the overall diagnostic outcome based on TPD.

By analyzing the impact of RM correction using the segmental uptake values as semiquantitative parameter, we observed a change of ≥5% in uptake value in one or more segments in 57 patients (69%). The mean difference in segmental uptake values varied between −2.0% and 2.1% points and seemed unrelated to territorial areas. The difference between the segmental uptake values of both noncorrected and RM-corrected scans seems to increase for lower average segmental uptake values, as shown in Figure 3B. Corrections in segments that already show perfusion defects can be considered as less important than segments which are corrected from or to normal which occurred less frequently. The ≥5% segmental uptake changes corresponded with FFR in 30 patients but were in discordance with 15 patients and remained unknown in 12 patients, in whom segmental defects both appeared and disappeared after RM correction. One or more segments were positively corrected with an uptake value of ≥5% in 23 patients, indicating a correction of a possible defect. In 17 of these 23 patients a negative FFR was found, which can be considered as an improvement but in the other six patients the correction was considered a deterioration. In 22 patients, the changed segments were corrected negatively, indicating the existence of a possible defect. In these 22 patients, a positive FFR was found in 13 patients. However, in the other nine of these 22 patients, a negative FFR was found, possibly indicating a deterioration after RM correction. In the twelve (14%) remaining patients, both positive and negative segmental uptake corrections of ≥5% were observed. So the correction resulted in the disappearance of one or more defects but in the originating of other defects. In eight of these 12 patients, a negative FFR was found and in four a positive FFR but it is unknown whether this was an improvement or deterioration. So RM correction resulted in 30 improvements but also in 15 deteriorations in the overall diagnostic outcomes based on segmental uptake values.

### Diagnostic Value of Patient Motion Correction

Applying PM correction resulted in less differences between the noncorrected and corrected scans. The diagnostic outcomes did not change in any of the patients based on SPECT interpretation or TPD after PM correction, as shown in Figures 2D and 3C. However, the diagnostic outcome was influenced in seven patients (8%) based on a ≥5% change in segmental uptake values, as shown in Figure 3D. The segmental uptake values were corrected positively for all seven patients, indicating possible corrections of defects. However, in only four patients a negative FFR was found, considered an improvement. In the other three patients, a positive FFR was found, indicating a deterioration of the diagnostic outcomes.

### Relation Between Motion Detection and Correction

The amount of motion correction and the mean RM or PM did not to correlate, as shown in Figure 2. The correlation between the mean RM and differences in SPECT interpretation, TPD or segmental uptake were not significant (*P* = .26, *P* = .65, and *P* = .27, respectively). This was also the case for mean PM and number of deviating segments (*P* = .13). As PM correction did not influence the diagnostic outcomes for SPECT interpretation or TPD, the correlation between PM and these variables was not derived.

## Discussion

In this study, we have shown that patients undergoing an 8-minute CZT-SPECT scan barely move, as both respiratory motion and patient motion were limited. Nevertheless, applying automatic motion correction changed the SPECT interpretation in 11% of the stress scans. However, these changes were both deteriorations and improvements which led us to conclude that motion correction did not seem to improve the overall diagnostic outcomes of CZT-SPECT. Moreover, motion correction also changed semiquantitative outcome parameters, such as TPD and segmental uptake values, but neither these changes could be considered an overall improvement, as compared to the FFR measurements.

Both the RM and PM measured in this study were smaller than reported in previous studies using the same CZT-based SPECT camera.^8^,^13^ Ko et al reported a mean RM of 10.5 mm in the cranial-caudal direction using a pharmaceutical stress agent, which is much larger than the mean RM of 2.5 mm as we encountered in the present study.^8^ The smaller motion in our study might be due to the following reasons: the use of longer timing bins in our study (1.0 second instead of 0.5 second); higher myocardial count rates due to the use of 99m-Technicium instead of Thallium-201, which decreases noise and increases the count statistics and resolution; and the exclusion of reproducibility errors in the masking and manual alignment prior to reconstruction. Although they performed a phantom study demonstrating the correctness of their motion tracking program, they filled their cardiac phantom with a fifth of the activity administered to their patients. Hence, they had far more count statistics during their phantom study than encountered in their patient studies. This might indicate that they detected more noise during their patient studies, possibly explaining the larger detected RM. They reported, based on their phantom study, that a RM larger than 15 mm could cause visual and quantitative image deterioration. However, this RM threshold was never reached in our study. Nevertheless, we applied RM correction in all patients, resulting in a changed SPECT interpretation in 11% of our scans. As we did not find a correlation between RM correction and changes in SPECT interpretation, TPD or segmental values, the changes we observed were possibly due to overcorrections by the automatic software because of inadequate count statistics. This is in agreement with a phantom study we performed in which we validated the motion detection software (this phantom study is described in more detail in the supplementary materials). A manually induced motion was detected within an uncertainty of typically 2 mm using 1-second time bins (corresponding to RM) and 1 mm using 20-second time bins (corresponding to PM). The maximum detection error was in the order of 8 mm for RM. Correction of the limited motion, within the margin of error, is therefore not expected to result in an improvement in the scan in relation to the diagnostic outcome. It may even lead to a deterioration of image quality. Hence, due to the limited detected motion, it is likely that RM correction is not useful in our patient population and application only resulted in overcorrections.

In contrast to the limited effect of correcting for respiratory motion in our study, a recent study by Clerc et al suggested that deleting respiratory motion by deep inspiration breath holding during MPI CZT-SPECT acquisition was beneficial.^25^ Breath holding resulted in 12.5% more normal scans in their 40 patients when also using attenuation correction. Acquisition during breath holding causes a caudal shift of the abdominal structures which may prevent inferior wall artefacts and improve co-registration with the inspiration breath-hold CT used for attenuation correction. However, the reported results have not been compared with a reference standard. In addition, starting and stopping the acquisition after each breath hold should be perfectly timed by the operators and be available on the SPECT system. Repeated long breath holding can also be quite difficult for patients and it may even require the administration of higher tracer activities in order to limit the length of total acquisition, which in turn raises radiation dose again.^26^,^27^

The detected PM in the present study was also lower than the PM reported by Redgate et al, who even used a shorter acquisition time of 6 minutes.^13^ Using data of 40 patients, they recently reported a mean PM of 0-4, 4-8, and ≥8 mm in 62%, 35%, and 3%, respectively. However, none of the 83 patients in our study had a mean PM larger than 4 mm. We only encountered a maximum PM of ≥8 mm in 2% (2) of the patients, in contrast to the 10% they reported. The lower PM in our study could be due to exclusion of reproducibility errors in the present study and the comfortable patient environment in combination with extensive patient preparation to calm the patients. Redgate et al concluded from their phantom study that PM should be corrected when it exceeds 10 mm for more than 60 seconds. The maximum PM encountered in this study was 8.9 mm in one time bin of 20 seconds and PM correction did not result in changed SPECT interpretation. Hence, these figures seem to confirm our finding that, similar to RM, PM correction is not necessary in our patient population.

Several assumptions underpinned this study. First, we used a retrospective cohort of patients; all referred for elective FFR measurements after invasive coronary angiography. The incidence of ischemia and irreversible defects was 4% and 11% higher in the present study, respectively, in comparison to what we commonly encounter in our population eligible for MPI CZT-SPECT.^28^ Although the incidence of perfusion defects was not expected to influence RM or PM, it induces lower count statistics, possibly resulting in a higher tracking—and therefore also correction—error.

Second, we used FFR as a reference standard to assess the added value of motion correction on the diagnostic outcome. Although the accuracy of FFR is limited in patients with collateral circulation or serial stenosis,^29^ it is nowadays considered as one of the most accurate tests to detect ischemia. We only compared the motion-corrected stress acquisitions with FFR, eliminating the possibility to distinguish reversible (ischemic) from irreversible defects as would have been possible when using both stress and rest acquisitions. Although occluded vessels were also interpreted as positive FFR, this might have led to a slight underestimation of the correspondence with FFR for negative motion corrections (normal scans corrected to equivocal or abnormal, or an increasing TPD or decreasing segmental values) and overestimation of positive motion corrections. Moreover, co-registration of MPI with coronary CT angiography was not performed. Therefore some of the changes in perfusion after RM correction may have occurred in a different coronary territory than the territory supplied by the vessel in which FFR measurement was done.^30^ Nevertheless, the discrepancies between FFR and MPI were considered to be too limited to influence the outcomes of this study.

Third, we only compared the motion-corrected scans with nonattenuation corrected scans. It was not possible to apply motion correction to attenuation corrected scans and as attenuation correction is not expected to compensate for motion, it would not have contributed to our aim.

Fourth, the percentage of patients in which motion correction changed the diagnostic outcomes differed between the three endpoints: SPECT interpretation, TPD, and segmental uptake values. Although the influence of motion correction seemed limited for all three endpoints, one should be cautious in future studies when using only one of these semiquantitative endpoints. It appears that a segmental uptake difference of 5% is a very sensitive parameter for a change in defects but not a very specific one in comparison to the SPECT interpretation, which is still the reference standard in most institutions. Moreover, using a TPD difference of 7% appears not sensitive enough in detecting change in perfusion deficits in comparison to the visual SPECT interpretation.

Finally, there is a current trend towards lower activities and patient-specific dose protocols.^31^,^32^ Lowering the activity can easily be achieved by enlarging the scan time, as both are interchangeable within a certain range. In this study, we showed that a scan time up to 8 minutes does not introduce significant motion. The motion even decreased in the first minutes. Using longer scan times in combination with lower activities might therefore even decrease the influence of the higher motion in the first minutes of the acquisition. However, one should be aware that correction of observed motion will be harder with lower count statistics and that acquisitions should be repeated instead of corrected. The amount of motion depends on the calmness and relaxedness of the patients. We think it is of great importance to create a comfortable patient environment, provide clear instructions and to provide extensive patient information prior to MPI to reduce motion.

## Conclusion

Both respiratory motion and patient motion were small in patients undergoing an 8-minute MPI acquisition on a CZT-based SPECT camera. Correction of this small motion did not appear to improve the diagnostic outcomes. Hence, the value of applying motion correction seems limited in MPI using a CZT-based SPECT camera.

## New Knowledge Gained

The respiratory motion and patient motion detected in this study by commercial software are lower than reported by previous studies using self-developed tracking algorithms. Correction of small motion did not appear to improve the diagnostic outcomes and, hence, the added value seems limited in 8-minute MPI acquisitions using a CZT-based SPECT camera.

## Electronic Supplementary Material

Below is the link to the electronic supplementary material.

Supplementary material 1 (DOCX 516 kb)

Supplementary material 2 (PPTX 414 kb)

## Abbreviations

CZT: Cadmium-zinc-telluride
FFR: Fractional flow reserve
MPI: Myocardial perfusion imaging
RM: Respiratory motion
PM: Patient motion
SPECT: Single photon emission computed tomography
TPD: Total perfusion deficit
