# Supplementary material for: Value of automatic patient motion detection and correction in myocardial perfusion imaging using a CZT-based SPECT camera
Source: J Nucl Cardiol. 2016 Jul 12;25(2):419–28. doi: 10.1007/s12350-016-0571-7 (PMC5869883; doi:10.1007/s12350-016-0571-7)
Supplement: Supplementary file 1 — Supplementary material 1 (DOCX 516 kb) [file 12350_2016_571_MOESM1_ESM.docx]

## Supplementary materials

### Accuracy of motion detection and correction software: a phantom study.

#### Aim

To determine the accuracy of automatic motion detection and correction software (MCD for Alcyone, GE Healthcare) using a phantom study.

#### Methods and materials

A phantom simulating the ventricular wall (Cardiac Insert^TM^ ) was filled with 20 MBq Tc-99m Tetrofosmin. Next, we placed this phantom in a larger phantom filled with water (Jaszczak Phantom™) to simulate attenuation. The phantom was placed in the CZT-SPECT camera (Discovery NM/CT 570c, GE Healthcare) and positioned in the center of the field of view, assisted by using real-time persistence imaging. Data were acquired in list mode for 180s using a 20% symmetrical energy window centered at 140 keV. Every 20s, the table was moved into another position to induce motion, as shown in table 1. Acquisition was paused during table movements.

Table 1. Table positions during the phantom acquisitions to simulate motion. Motion was simulated in the cranial-caudal direction (z) and the anterior posterior direction (y).

| Time | Induced motion |
| --- | --- |
| 0-20s | 0 mm (z) |
| 21-40s | -15 mm (z) |
| 41-60s | -10 mm (z) |
| 61-80s | -5 mm (z) |
| 81-100s | +5 mm (z) |
| 101-120s | +10 mm (z) |
| 121-140s | +15 mm (z) |
| 141-160s | +5 mm (z) -5mm y |
| 161-180s | -5 mm (z) +5mm y |

#### Results

The count rates in this phantom study were similar to those encountered in clinical practice. The average deviation between the measured motion and the induced motion was -0.9 ± 2.4 mm when using 1s time bins (corresponding to respiratory motion (RM) detection), as shown in Figure 1. Hence, a systematic error of the MDC software to detect motion is within 1 mm. However, the average absolute deviation between the detected motion and induced motion was 2.1 ± 1.6 mm (range: 0.0-8.4mm) in the cranial-caudal direction for 1s time bins. This higher absolute error is due to the noise as a result of statistical variation when using shorter time bins with limited count statistics, as shown in Figure 1. The MDC software corrects for motion in each time bin. Outliers as a result of noise are therefore also corrected, possibly explaining the deterioration of image quality after applying motion correction in our patient study.

The average deviation between the detected motion and induced motion was -1.1 ± 0.8, 0.9 ± 0.9 and 0.0 ± 1.0 mm for the lateral, anterior-posterior and cranial-caudal motion, respectively, when using 20s time bins, as shown in Figure 2. This indicates a possible systematic bias of 1.1 mm. The average absolute deviation was 1.1 ± 0.8 mm, 1.1 ± 0.8 mm and 0.8 ± 0.6 mm for the lateral, anterior-posterior and cranial-caudal motion, respectively. This absolute deviation is lower than when using 1s time bins due to the 20-fold larger bin size resulting in increased count statistics and consequently decreased statistical fluctuations. Due to the limited statistical error component using 20s bins, correction of limited motion is more appropriate than when using the 1s bins. These findings support the results of our patient study, demonstrating the limited effect on the diagnostic outcome of applying PM correction in case of limited motion.


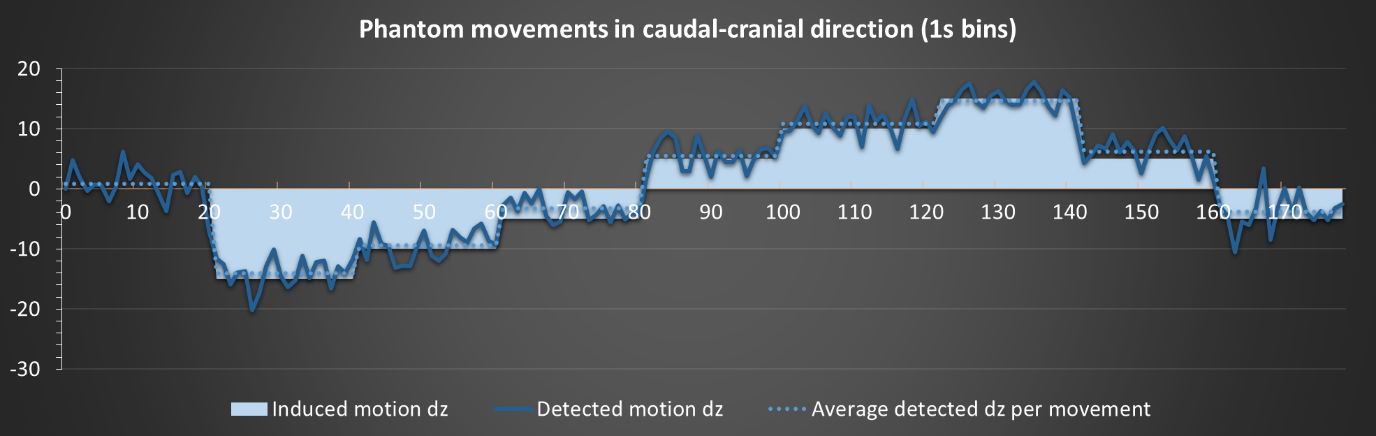


**Figure 1.** The induced cranial caudal motion (blue area) and the detected motion by the MDC software using 1s time bins (blue line). The dotted line represents the average detected motion for each table position (averaged over 20s). In the ideal situation, the blue line would exactly describe the edges of the shaded area.


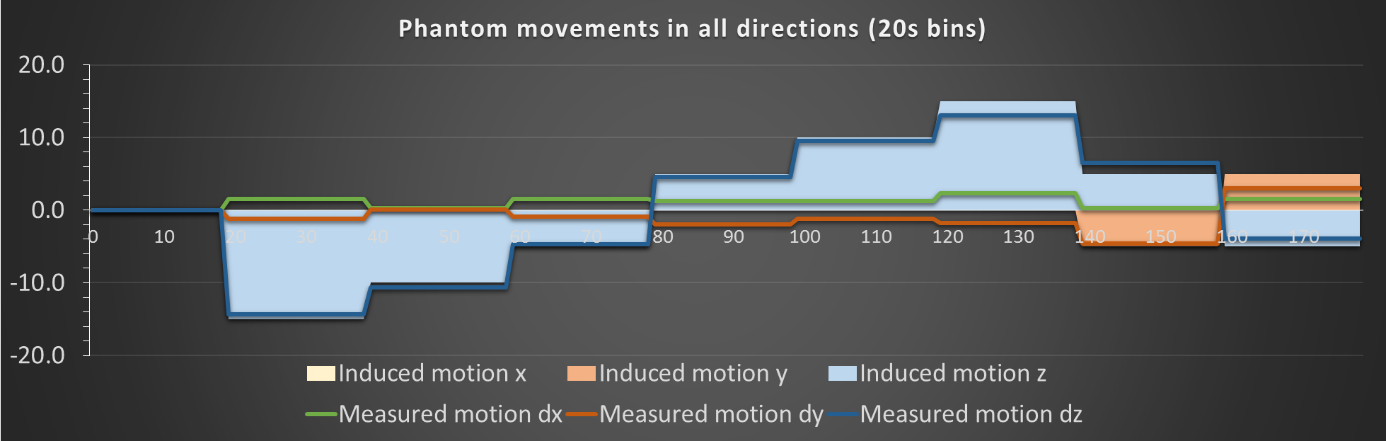


**Figure 2.** The induced cranial-caudal motion (blue area) and anterior-posterior motion (orange area) and the detected motion using 20s time bins in the x (lateral), y (anterior-posterior) and z (cranial-caudal) directions by the MDC software, represented by the green, orange and blue lines, respectively.

#### Conclusion

MDC software can accurately detect a mean respiratory motion larger than typically 2mm. The software is able to detected patient motion larger than typically 1mm. Limited detected motion can be the result of a statistical error, due to insufficient count statistics. Correction of this non-existing motion, especially when using smaller time bins with less count statistics, might result in a deterioration of the image quality. Hence, although the software is able identify respiratory motion and patient motion larger than 2 or 1 mm, respectively, correction of limited motion may result in deterioration of image quality.
