# Supplementary material for: Value of automatic patient motion detection and correction in myocardial perfusion imaging using a CZT-based SPECT camera
Source: J Nucl Cardiol. 2016 Jul 12;25(2):419–28. doi: 10.1007/s12350-016-0571-7 (PMC5869883; doi:10.1007/s12350-016-0571-7)
Supplement: Supplementary file 2 — Supplementary material 2 (PPTX 414 kb) [file 12350_2016_571_MOESM2_ESM.pptx]

## Slide 1
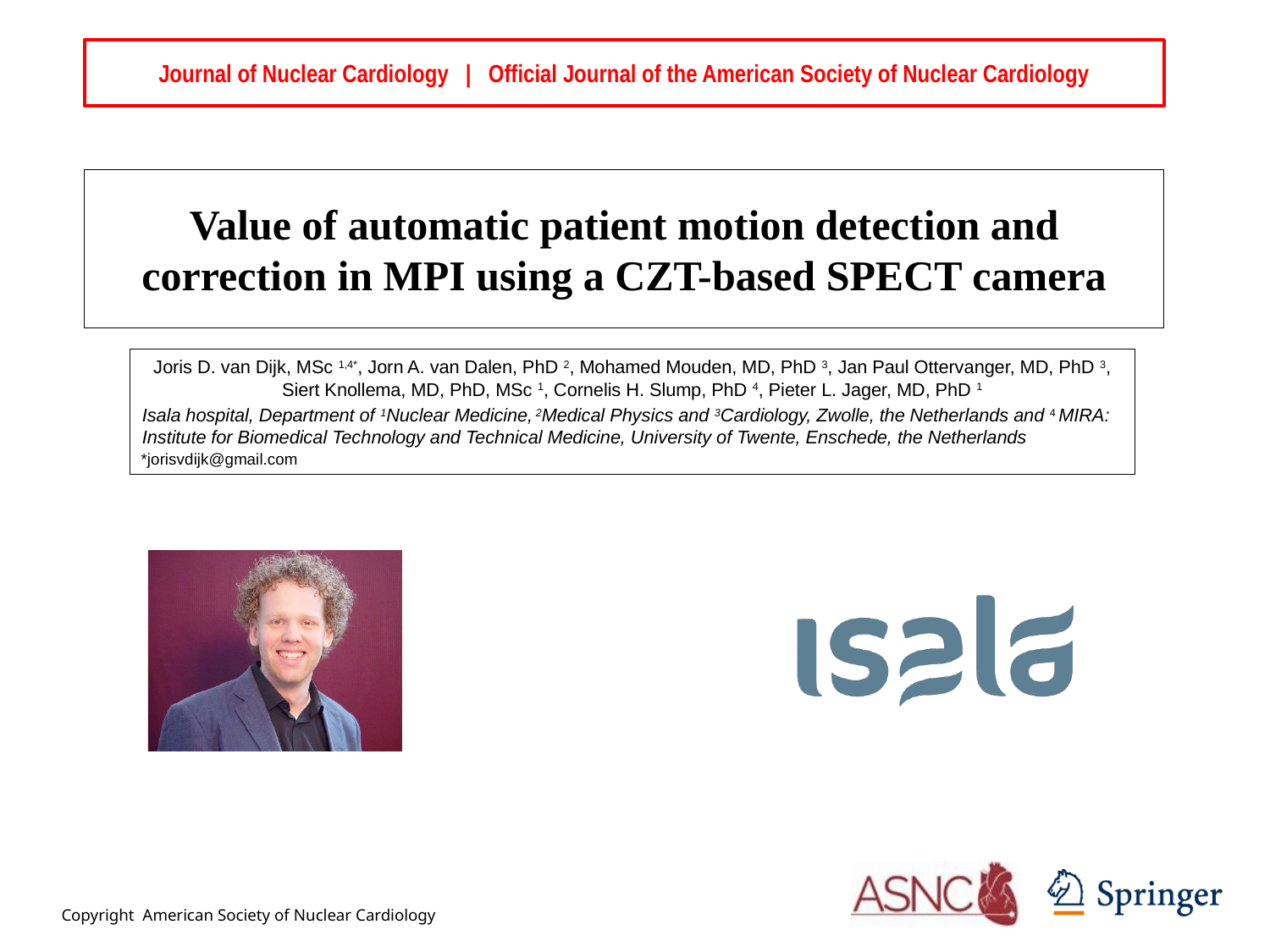

Journal of Nuclear Cardiology | Official Journal of the American Society of Nuclear Cardiology
# Value of automatic patient motion detection and correction in MPI using a CZT-based SPECT camera
Joris D. van Dijk, MSc 1,4*, Jorn A. van Dalen, PhD 2, Mohamed Mouden, MD, PhD 3, Jan Paul Ottervanger, MD, PhD 3, Siert Knollema, MD, PhD, MSc 1, Cornelis H. Slump, PhD 4, Pieter L. Jager, MD, PhD 1
Isala hospital, Department of 1Nuclear Medicine, 2Medical Physics and 3Cardiology, Zwolle, the Netherlands and 4 MIRA: Institute for Biomedical Technology and Technical Medicine, University of Twente, Enschede, the Netherlands
*jorisvdijk@gmail.com
Copyright American Society of Nuclear Cardiology

## Slide 2
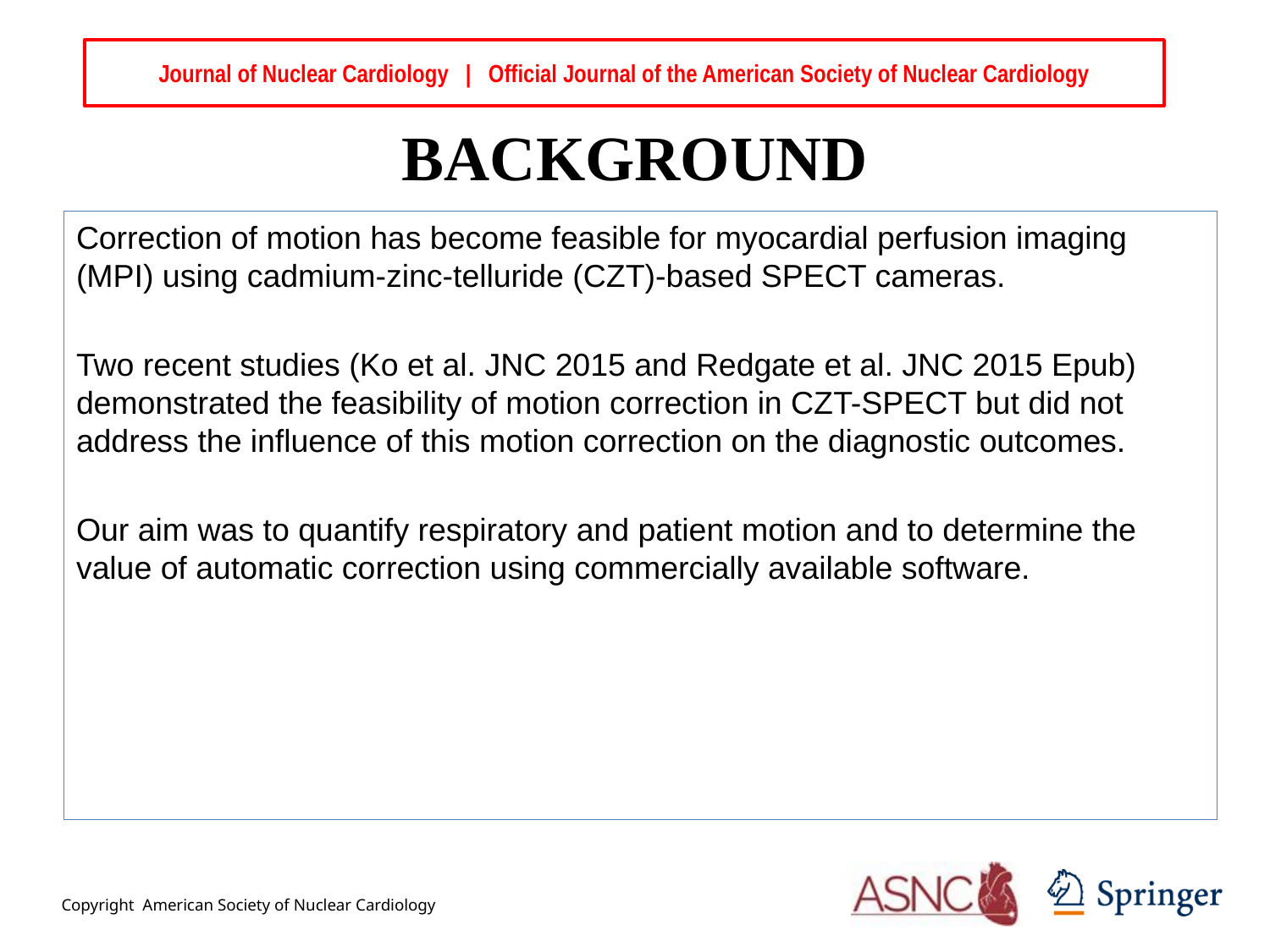

Journal of Nuclear Cardiology | Official Journal of the American Society of Nuclear Cardiology
# BACKGROUND
Correction of motion has become feasible for myocardial perfusion imaging (MPI) using cadmium-zinc-telluride (CZT)-based SPECT cameras.
Two recent studies (Ko et al. JNC 2015 and Redgate et al. JNC 2015 Epub) demonstrated the feasibility of motion correction in CZT-SPECT but did not address the influence of this motion correction on the diagnostic outcomes.
Our aim was to quantify respiratory and patient motion and to determine the value of automatic correction using commercially available software.
Copyright American Society of Nuclear Cardiology

## Slide 3
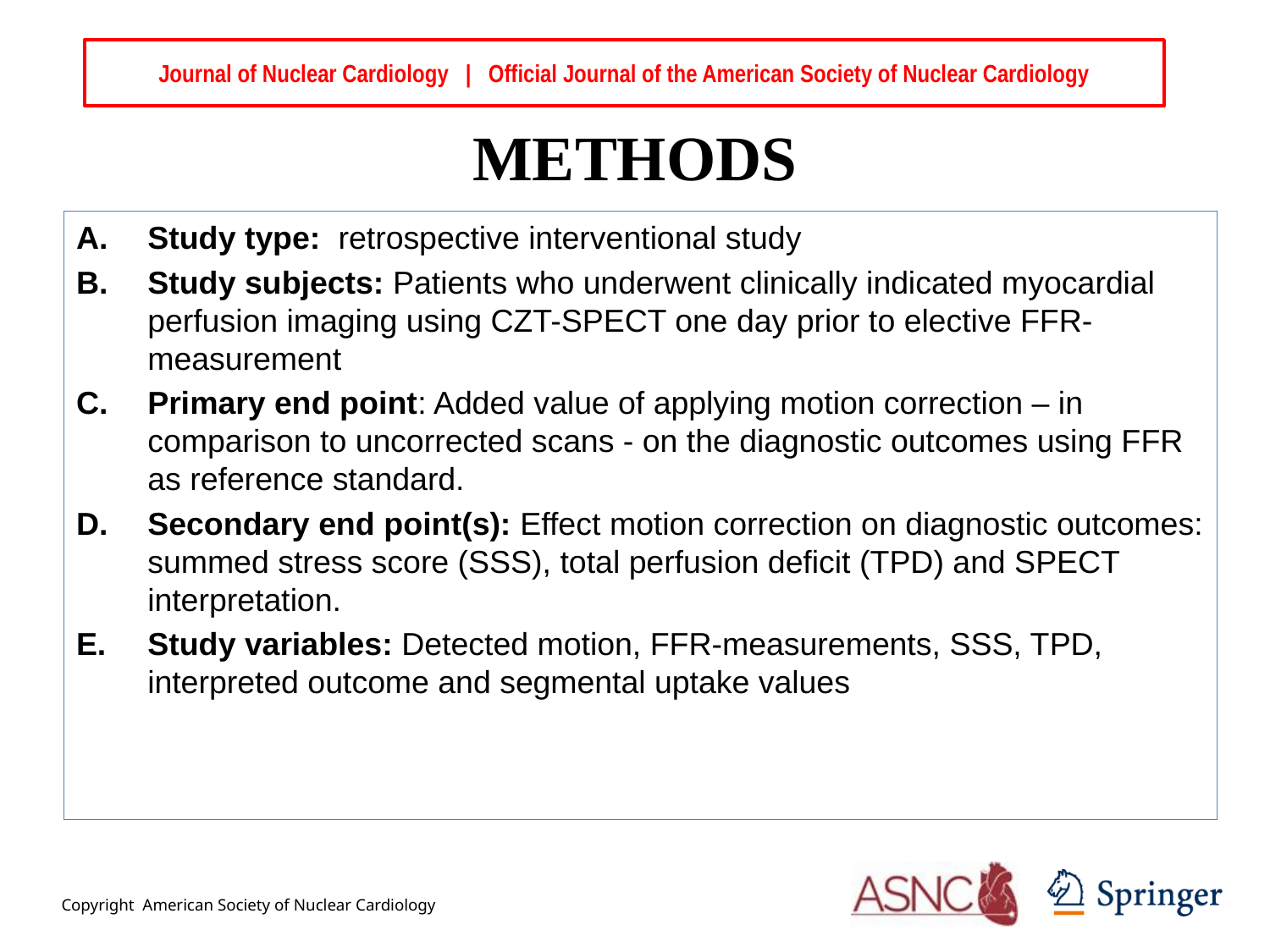

Journal of Nuclear Cardiology | Official Journal of the American Society of Nuclear Cardiology
# METHODS
Study type: retrospective interventional study
Study subjects: Patients who underwent clinically indicated myocardial perfusion imaging using CZT-SPECT one day prior to elective FFR-measurement
Primary end point: Added value of applying motion correction – in comparison to uncorrected scans - on the diagnostic outcomes using FFR as reference standard.
Secondary end point(s): Effect motion correction on diagnostic outcomes: summed stress score (SSS), total perfusion deficit (TPD) and SPECT interpretation.
Study variables:	Detected motion, FFR-measurements, SSS, TPD, interpreted outcome and segmental uptake values
Copyright American Society of Nuclear Cardiology

## Slide 4
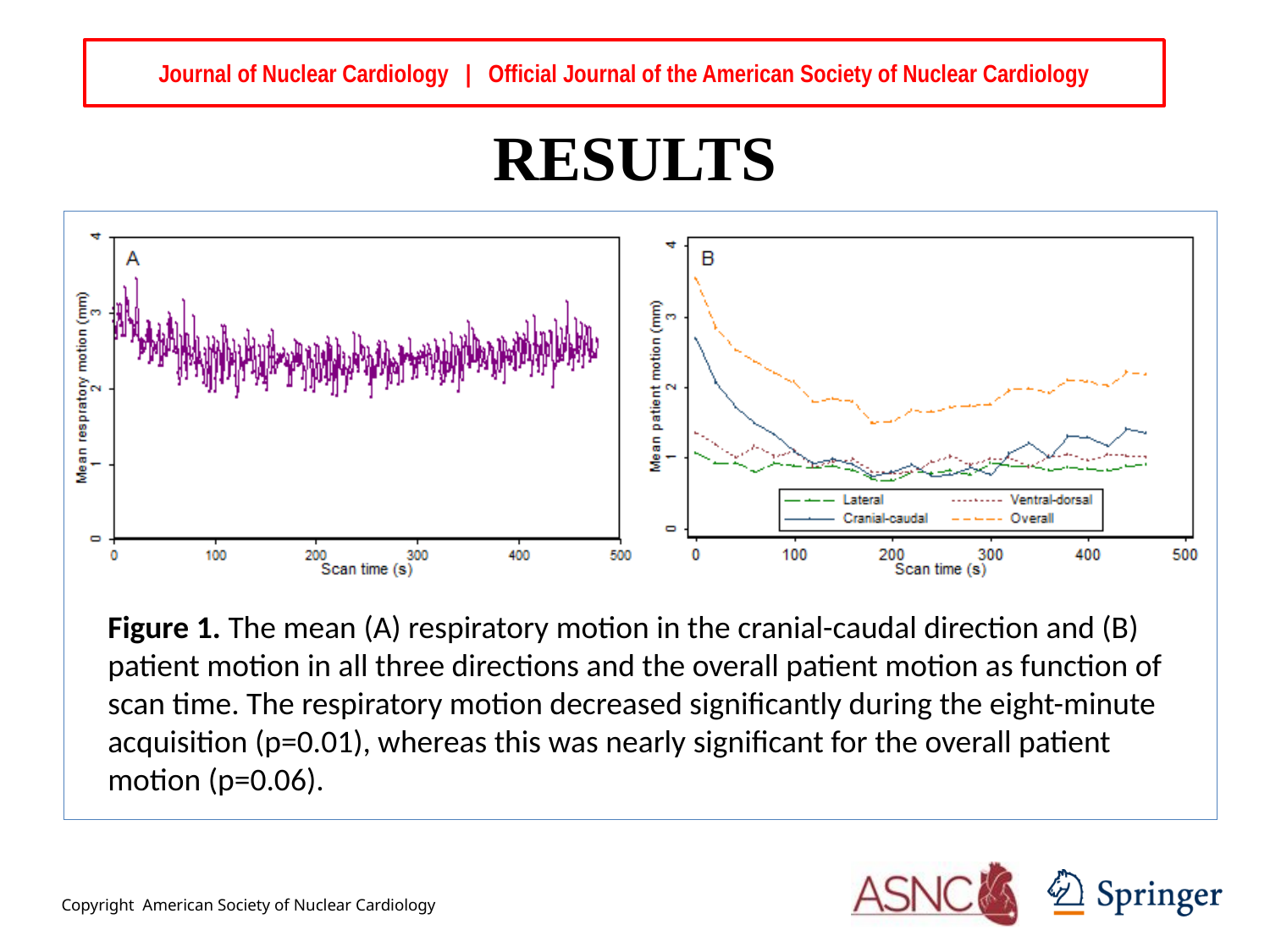

Journal of Nuclear Cardiology | Official Journal of the American Society of Nuclear Cardiology
# RESULTS
Figure 1. The mean (A) respiratory motion in the cranial-caudal direction and (B) patient motion in all three directions and the overall patient motion as function of scan time. The respiratory motion decreased significantly during the eight-minute acquisition (p=0.01), whereas this was nearly significant for the overall patient motion (p=0.06).
Copyright American Society of Nuclear Cardiology

## Slide 5
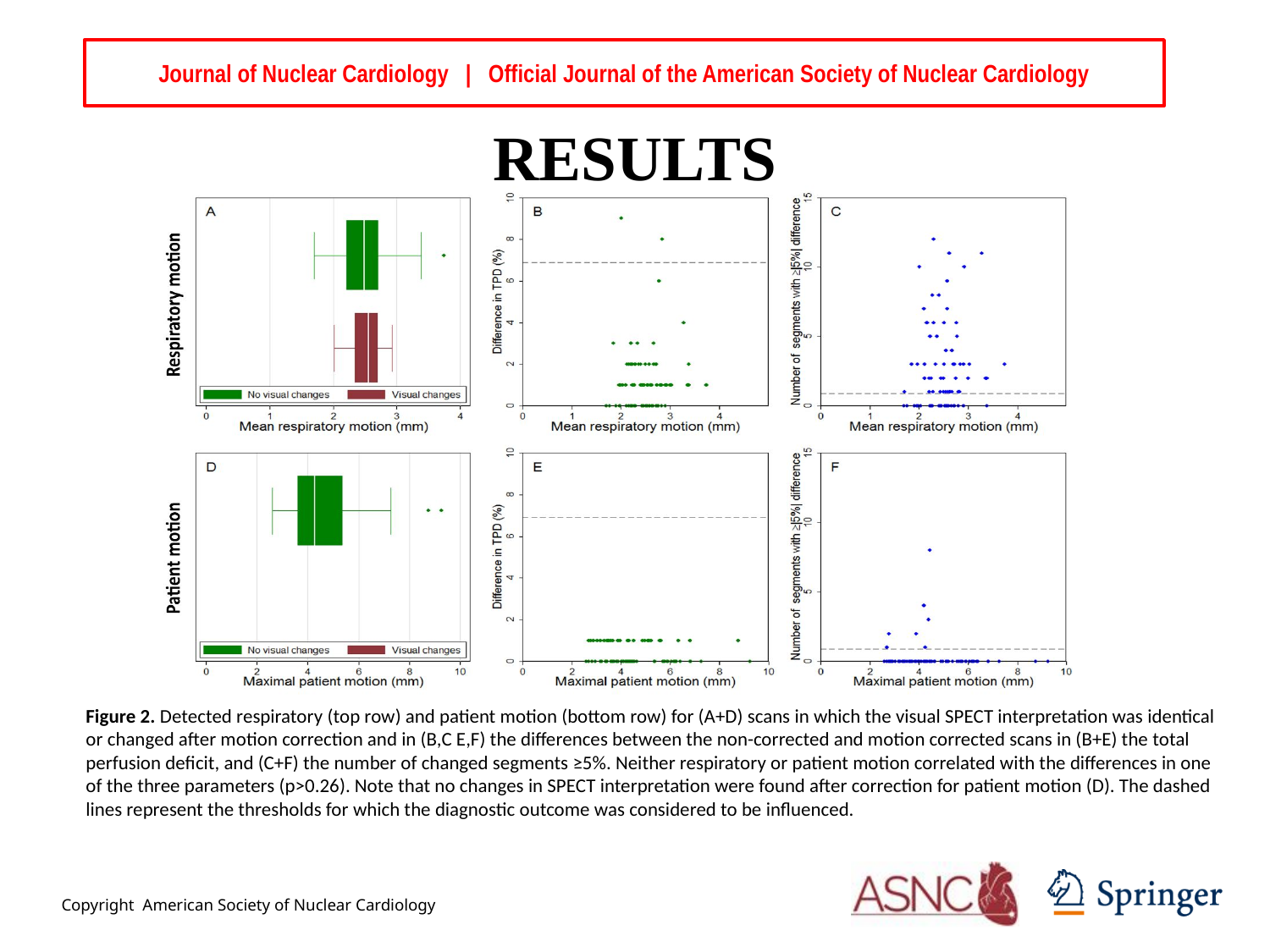

Journal of Nuclear Cardiology | Official Journal of the American Society of Nuclear Cardiology
# RESULTS
Figure 2. Detected respiratory (top row) and patient motion (bottom row) for (A+D) scans in which the visual SPECT interpretation was identical or changed after motion correction and in (B,C E,F) the differences between the non-corrected and motion corrected scans in (B+E) the total perfusion deficit, and (C+F) the number of changed segments ≥5%. Neither respiratory or patient motion correlated with the differences in one of the three parameters (p>0.26). Note that no changes in SPECT interpretation were found after correction for patient motion (D). The dashed lines represent the thresholds for which the diagnostic outcome was considered to be influenced.
Copyright American Society of Nuclear Cardiology

## Slide 6
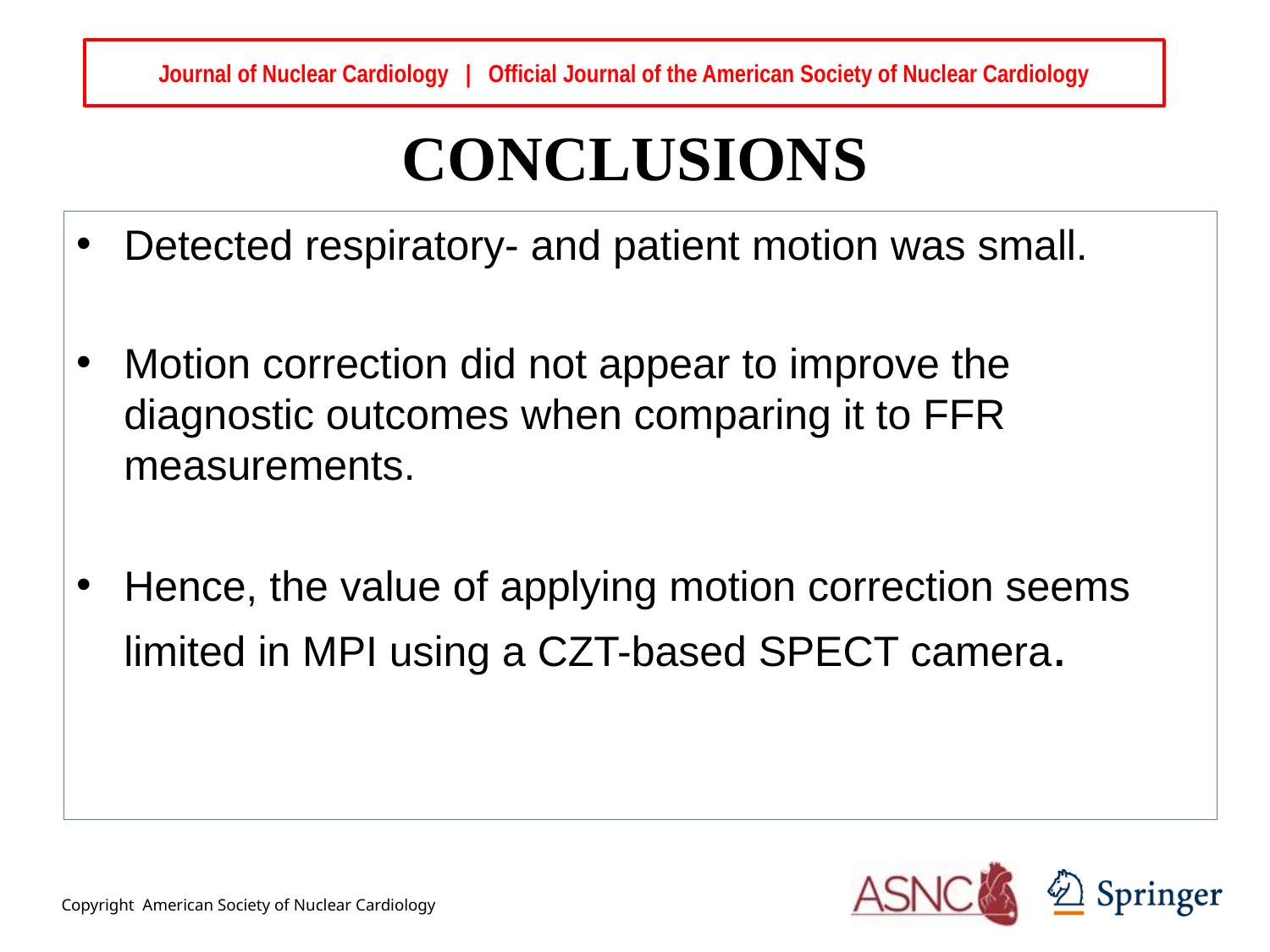

Journal of Nuclear Cardiology | Official Journal of the American Society of Nuclear Cardiology
# CONCLUSIONS
Detected respiratory- and patient motion was small.
Motion correction did not appear to improve the diagnostic outcomes when comparing it to FFR measurements.
Hence, the value of applying motion correction seems limited in MPI using a CZT-based SPECT camera.
Copyright American Society of Nuclear Cardiology
